# The Associations of Youth Physical Activity and Screen Time with Fatness and Fitness: The 2012 NHANES National Youth Fitness Survey

**DOI:** 10.1371/journal.pone.0148038

**Published:** 2016-01-28

**Authors:** Yang Bai, Senlin Chen, Kelly R. Laurson, Youngwon Kim, Pedro F. Saint-Maurice, Gregory J. Welk

**Affiliations:** 1 Department of Kinesiology, Iowa State University, Ames, Iowa, United States of America; 2 School of Kinesiology and Recreation, Illinois State University, Normal, Illinois, United States of America; 3 MRC Epidemiology Unit, University of Cambridge, Cambridge, United Kingdom; Stanford University School of Medicine, UNITED STATES

## Abstract

The purpose of the study is to examine the associations of youth physical activity and screen time with weight status and cardiorespiratory fitness in children and adolescents, separately, utilizing a nationally representative sample. A total of 1,113 participants (692 children aged 6–11 yrs; 422 adolescents aged 12–15 yrs) from the 2012 NHANES National Youth Fitness Survey. Participants completed physical activity and screen time questionnaires, and their body mass index and cardiorespiratory fitness (adolescents only) were assessed. Adolescents completed additional physical activity questions to estimate daily MET minutes. Children not meeting the screen time guideline had 1.69 times the odds of being overweight/obese compared to those meeting the screen time guideline, after adjusting for physical activity and other control variables. Among adolescent, screen time was significantly associated with being overweight/obese (odds ratio = 1.82, 95% confidence interval: 1.06–3.15), but the association attenuated toward the borderline of being significant after controlling for physical activity. Being physically active was positively associated with cardiorespiratory fitness, independent of screen time among adolescents. In joint association analysis, children who did not meet physical activity nor screen time guidelines had 2.52 times higher odds of being overweight/obese than children who met both guidelines. Adolescents who did not meet the screen time guideline had significantly higher odds ratio of being overweight/obese regardless of meeting the physical activity guideline. Meeting the physical activity guideline was also associated with cardiorespiratory fitness regardless of meeting the screen time guideline in adolescents. Screen time is a stronger factor than physical activity in predicting weight status in both children and adolescents, and only physical activity is strongly associated with cardiorespiratory fitness in adolescents.

## Introduction

Established evidence documents that low levels of physical activity (PA) increase risk for obesity and other negative health outcomes in youth [1–3]. Excessive time spent in sedentary behavior, especially screen-based sedentary behavior, has emerged as another important, modifiable risk factor [4–6]; however, it has proven difficult to isolate the independent effects of PA and screen-based sedentary behavior on negative health outcomes due to differences in study samples, and methodologies [7] between studies. Some studies have suggested that the type of sedentary behavior, such as screen time (ST), might be more important than overall sedentary time in relation to youth health [8–11]. In addition, Saunders et al. pointed out in a recent review that future research is particularly needed to investigate the impact of characteristics of sedentary behavior (i.e., type and context) [7]. Several well-designed studies [1, 12] demonstrated that low levels of PA were strongly associated with obesity, independent of ST, but mixed results were found for the association between ST and obesity when adjusting for PA [7, 9, 13, 14].

In addition, limited evidence is available regarding the independent and joint effects of PA and/or ST, on cardiorespiratory fitness (CRF), another critical indicator of youth health [15–17]. A review conducted by Parikh [18] concluded that vigorous PA is significantly associated with CRF, but the independent effects of screen-based sedentary behavior on CRF remain unclear [19–22]. The impact of ST on youth CRF may be less substantial than it is on body weight, but further exploration is required.

Unraveling the complex influence of PA and screen-based sedentary behavior and their interplay on fitness and fatness will provide unique insights to public health and education professionals focused on promoting health in youth. The lack of updated nationwide youth fitness data since the early 2000’s has limited the ability to explore the independent, as well as the joint associations of PA and ST with fitness in children and adolescents. The recent release of data from the NHANES National Youth Fitness Survey (NNYFS) provides a unique opportunity for researchers to systematically study this relation with a national representative sample. The NNYFS thoroughly surveyed the contextual characteristics of youth PA and ST. Thus, the purpose of the current study is to determine the independent and joint associations of PA and ST with weight status and CRF in the NNYFS.

## Materials and Methods

### Study design and population

The data are from the 2012 NNYFS conducted by the Centers for Disease Control and Prevention National Center for Health Statistics (NCHS). The NNYFS compiles fitness and health indicators from a nationally representative sample of fitness and health in 3–15 years old non-institutionalized U.S. youth. Measurements consisted of a household interview followed by an assessment of PA and health-related fitness components conducted in a Mobile Examination Center (MEC). The NCHS Ethics Review Board reviewed and approved NNYFS protocols and written informed consent was obtained from all participants and/or their parents or guardian. A total of 1,576 children and adolescents (aged 6 to 15 yrs) completed the interview and MEC assessments. Consistent with recommendations from the NCHS, 6 to 11 yr-old participants were categorized as children, and 12 to 15 yr-old as adolescents.

### Outcome measures

The participants were measured for standing height using a stadiometer SECA 217 and body weight using portable scale SECA 869 (Chino, CA, USA). Body mass index was expressed as weight in kilograms divided by height in meters squared (kg/m^2^). Weight status was classified into underweight, normal weight, overweight, and obese based on the age-and gender-specific 5th, 85th, and 95th percentiles of the 2000 CDC growth charts cutoff points [23]. In the current study, underweight and normal weight (NW) were combined as normal weight (weight status = 0), and overweight and obese (OW/OB) were combined as overweight/obese (weight status = 1).

Standard submaximal treadmill test was used to assess CRF in past NHANES surveys (adolescents only) [24]. Age- and gender- specific health-related criterion-referenced standards from the FITNESSGRAM were used to evaluate the participants’ CRF levels [25]. Participants were specifically categorized into either the “Healthy Fitness Zone” (CRF = 0) or the “Needs Improvement Zone” (CRF = 1)

### Exposure measures

PA and ST were measured with a self-reported questionnaire during the household interview. The proxy respondents answered the questions for participants’ aged 6–11 yrs. PA was assessed by a single question in which participants reported the number of days in the past week that they were physically active (i.e., accumulated moderate and vigorous PA) for a total of at least 60 minutes. PA was recoded into a dichotomized variable of children and adolescents either “Meeting the PA Guidelines (i.e. 7days) or “Not Meeting the PA Guidelines (i.e. <7 days).

ST was assessed with two questions which prompted the participants to recall “Over the past 30 days, on average how many hours per day did you sit and watched TV or videos”, and “Over the past 30 days, on average how many hours per day did you use a computer or play computer games outside of work or school”. Responses ranged from 0 (< 1 hour) to 5 (≥5 hours) in in 1-hour increments except “< 1 hour” which was coded as 0.5. In our analysis, we combined the scores from the two ST questions and categorized children and adolescents into two groups based on total ST. Public health recommendations call for youth to limit daily ST to ≤ 2 hours [26, 27] so the two levels for children and adolescents were 1) Meeting the ST Guidelines (≤ 2 hours/day) and 2) Not Meeting the ST Guidelines (>2 hours/day).

Adolescents were asked to complete additional questions designed to capture time spent in moderate PA (MPA) and vigorous PA (VPA) in three settings (Work, Recreation, and Transportation). Time estimates were converted to total weekly MET-minutes (METmin) using NNYFS suggested MET scores (MPA ≥ 4 METS; Transportation = 4 METS; VPA ≥ 8 METS). Because of the relatively small sample size for adolescents, two approximately equal groups were created using the 50^th^ percentile of the MET-Minute values (i.e., a high active group and a low active group). The breakpoints for the high and low active groups were represented by ≥ 457 METmin per day, and < 457 METmin per day, respectively.

### Confounders

Potential confounders including age, gender, race/ethnicity, and socioeconomic status (SES) were controlled for in our analysis, based on previous evidence [28]. Ethnicity was categorized as non-Hispanic whites, non-Hispanic blacks, Hispanic, and “other”, based on the NCHS recommendations [24]. In the present study, SES was obtained by dividing family income by a poverty measure in accordance with established 2012 Department of Health and Human Services poverty guidelines. The sample’s SES ranged from 0 to 4.99 and a higher value indicated higher SES.

### Statistical analysis

Descriptive statistics for key variables were computed separately for children and adolescents. Crosstab of compliance with PA and ST guidelines was computed. Multiple logistic regressions were performed for each age group to examine the independent associations of the two exposure measures (i.e., PA and ST) with the two outcome measures (weight status and CRF). To further decipher the independent associations of PA and ST on weight status and CRF, when one variable was used as the predictor in the model, the other variable was entered as confounder to mutually adjust for each in the models. For example, PA was adjusted for ST when PA was entered as the main exposure variable, likewise ST was adjusted for PA when ST was entered as the main predictor. The same analyses were repeated by replacing PA with METmin to test associations with this more robust measure in the adolescent group.

Joint associations of PA and ST with weight status and CRF were evaluated with additional multiple logistic regression models. Combination exposure groups were created for both children and adolescents by combining PA and ST groups (i.e. Meet PA/Meet ST, Meet PA/Do Not Meet ST, Do Not Meet ST/Meet PA, and Do Not Meet PA/Do Not Meet ST). The meeting both PA and ST guidelines group was defined as the reference group in the joint analyses.

The logistic regression analyses adjusted for potential confounding demographic variables (e.g. age, gender, SES, and ethnicity) as well as other outcomes (e.g. CRF for weight status). Sampling weights in the MEC provided by NCHS were used to take into account selection probabilities, non-response, and non-coverage of the survey. Taylor series linearization was applied to estimate the variance of model parameters. All analyses were conducted with SAS 9.3 (Cary, NC) and SAS-Callable SUDAAN 11 (Raleigh, NC) software. The alpha value for significance testing was set at 0.05.

## Results

A total of 692 children and 422 adolescents with complete examination and questionnaire data were included in this study. The characteristics of the participants along with the levels of PA and ST are reported separately for children and adolescents groups in Table 1. Overall, sample sizes for boys and girls were evenly distributed. Non-Hispanic whites (37.1% and 45.7% for children and adolescents, respectively) were the most prevalent ethnic group, followed sequentially by Hispanic, non-Hispanic blacks, and others. More than one third of the participants were overweight or obese. Adolescents accumulated more daily ST and lower PA levels than children. Only approximately 3 in 10 children and 1 in 10 adolescents met both the PA and ST guidelines, while 3 in 10 children and 5 in 10 adolescents did not meet either the PA or ST guideline.

**Table 1:** Descriptive statistics of sample characteristics (unweighted estimates) in the 2012 NNYFS.

	Children (6–11 yrs)		Adolescents (12–15 yrs)	
Factors	N	%	N	%
**Sample Size**				
Total	692		422	
Boys	338	48.8%	218	51.7%
Girls	354	51.2%	204	48.3%
**Ethnicity**				
Non-Hispanic White	257	37.1%	193	45.7%
Non-Hispanic Black	164	23.7%	93	22.0%
Hispanic	210	30.4%	107	25.4%
Other	61	8.8%	29	6.9%
**Weight Status**				
Normal Weight	430	62.1%	263	62.3%
Over Weight	121	17.5%	74	17.6%
Obese	141	20.4%	85	20.1%
**Screen time**				
Meeting ST guideline	277	40.0%	116	27.5%
Not Meeting ST guideline	415	60.0%	306	72.5%
**PA**				
Meeting PA guideline	410	59.3%	116	27.5%
Not Meeting PA guideline	282	40.7%	306	72.5%
**Combined guideline**				
Both	192	27.8%	41	9.7%
Only PA	218	31.5%	75	17.8%
Only ST	85	12.3%	75	17.8%
Neither	197	28.5%	231	54.7%
**MET Minutes per day**				
High active			212	50.0%
Low active			210	50.0%

Abbreviations- PA: Physical Activity. Mod: Moderate

Table 2 shows the odds ratios from the multiple logistic regression models. Compared to children who met the PA guideline, children who did not meet the PA guideline had 1.65 times the odds of being OW/OB (See Model 1). Children who did not meet the ST guideline had 1.79 times the odds of being OW/OB compared to their peers who reported ≤2 hours of ST per day (See Model 2). After mutually adjusting for PA and ST in each model (See Model 3), meeting the PA guideline was no longer significantly associated with weight status in children, but the not meeting ST guideline group had 69% higher odds of being OW/OB compared to the low ST group.

**Table 2:** Logistic regression analysis of association between physical activity and screen time with weight status and Cardiorespiratory Fitness (CRF) in children and adolescents in the 2012 NNYFS.

	Children (6–11 yrs)		Adolescents (12–15 yrs)			
	Weight Status		Weight Status		CRF	
	OR	95% CI	OR	95% CI	OR	95% CI
**Model 1**						
***PA***						
Meeting PA guideline	1.0 (ref.)		1.0 (ref.)		1.0 (ref.)	
Not Meeting PA guideline	1.65	(1.03, 2.64) *	1.57	(0.82, 3.00)	1.8	(1.17, 2.78) *
**Model 2**						
***Screen time***						
Meeting ST guideline	1.0 (ref.)		1.0 (ref.)		1.0 (ref.)	
Not Meeting ST guideline	1.79	(1.20, 2.68) *	1.82	(1.06, 3.15) *	1.71	(0.74, 3.94)
**Model 3**						
***PA***						
Meeting PA guideline	1.0 (ref.)		1.0 (ref.)		1.0 (ref.)	
Not Meeting PA guideline	1.53	(0.92, 2.53)	1.44	(0.75, 2.75)	1.68	(1.15, 2.46) *
***Screen time***						
Meeting ST guideline	1.0 (ref.)		1.0 (ref.)		1.0 (ref.)	
Not Meeting ST guideline	1.69	(1.08, 2.65) *	1.72	(0.98, 3.00)	1.57	(0.70, 3.54)

* denotes significant association (P < 0.05) compared to the reference category. All the models were adjusted for age, gender, race/ethnicity, and socioeconomic status. Models used Weight Status as the outcome were also adjusted for CRF.

Abbreviations—PA: Physical Activity; CRF: Cardiorespiratory Fitness; OR: odds ratio; Mod: moderate.

Parallel analyses were carried out among adolescents for both the weight status and CRF outcome variables. For weight status, meeting the PA guideline was not associated with the odds of being OW/OB, but not meeting the ST guideline (> 2 hours per day) was found to be significantly associated with higher odds to be OW/OB. However, the significance disappeared after adjusting for PA (see Model 3). For CRF, meeting the PA guideline was significantly associated with high CRF and this remained significant after controlling for ST. Time spent in ST was not associated with CRF.

The independent associations of METmin and meeting the ST guideline with weight status and CRF were also tested in the adolescent sample (See Table 3). Participants with low daily METmin (< 457 METS) had significantly higher odds of being OW/OB and having low CRF. After controlling for ST, these associations remained statistically significant in the CRF model, but not in the weight status model.

**Table 3:** Logistic regression analysis of associations between MET minutes (METmin) and screen time with Weight Status and Cardiorespiratory Fitness (CRF) in adolescents in the 2012 NNYFS.

	Weight Status		CRF	
	OR	95% CI	OR	95% CI
**Model 1**				
**METmin**				
High active	1.0 (ref.)		1.0 (ref.)	
Low active	1.8	(1.07, 3.02) *	2.52	(1.31, 4.86) *
**Model 2**				
**METmin**				
High active	1.0 (ref.)		1.0 (ref.)	
Low active	1.66	(0.94, 2.91)	2.37	(1.29, 4.33) *
**Screen time**				
Meeting ST guideline	1.0 (ref.)		1.0 (ref.)	
Not Meeting ST guideline	1.64	(0.89, 3.01)	1.42	(0.68, 2.99)

* denotes significant association (P < 0.05) compared to the reference category. All the models were adjusted for age, gender, race/ethnicity, and socioeconomic status. Models used Weight Status as the outcome were also adjusted for CRF.

Abbreviations—BMI: Body Mass Index; CRF: Cardiorespiratory Fitness; OR: odds ratio; Mod: moderate.

**Fig 1:**
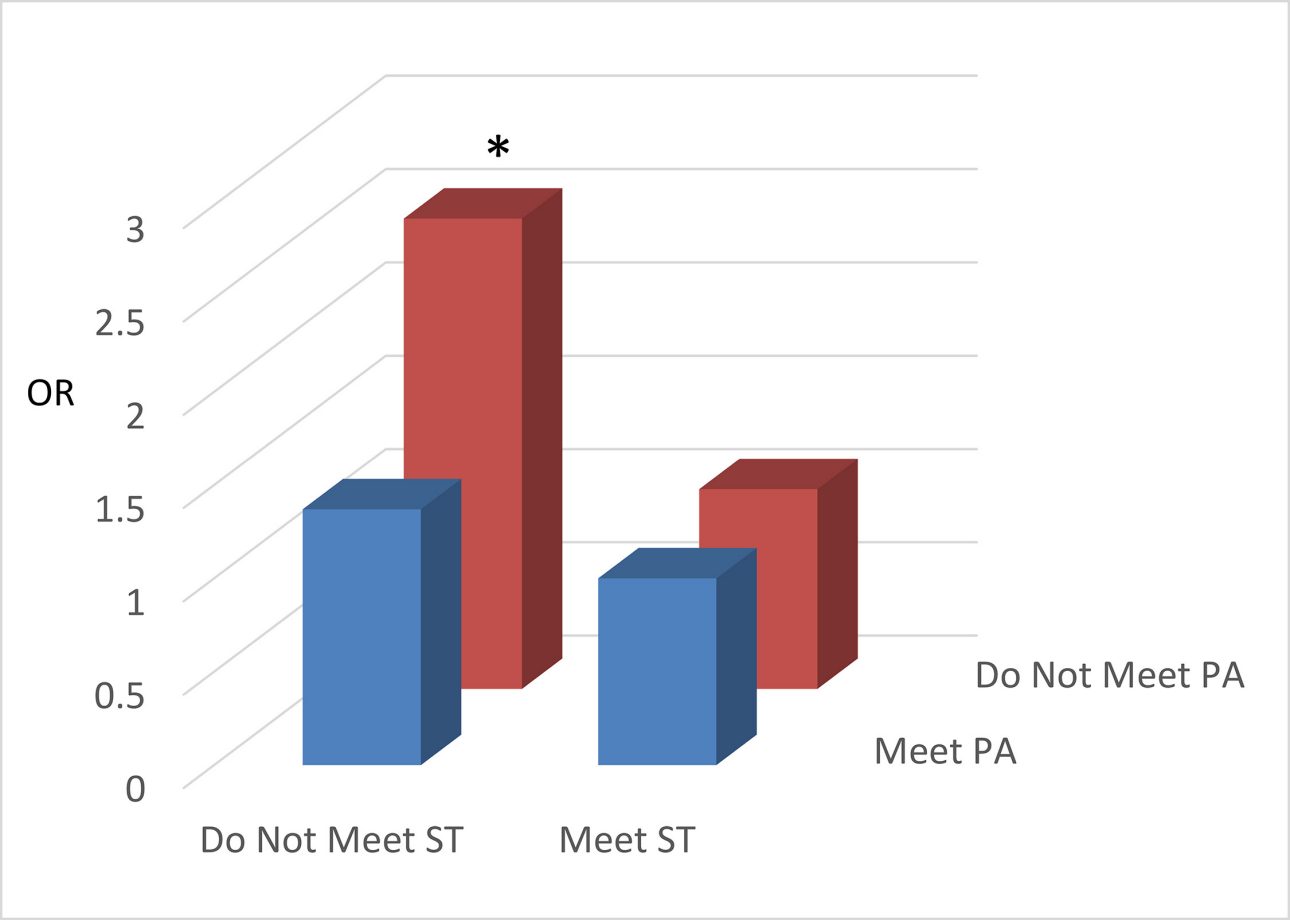
The joint associations of physical activity and screen time with weight status in children. All the odds ratio were adjusted for age, gender, race/ethnicity, and socioeconomic status. * denotes significant association (P < 0.05) compared to the reference category (Meet PA/Meet ST). Abbreviations–OR: Odds Ratio; PA: Physical Activity; ST: Screen Time.

**Fig 2:**
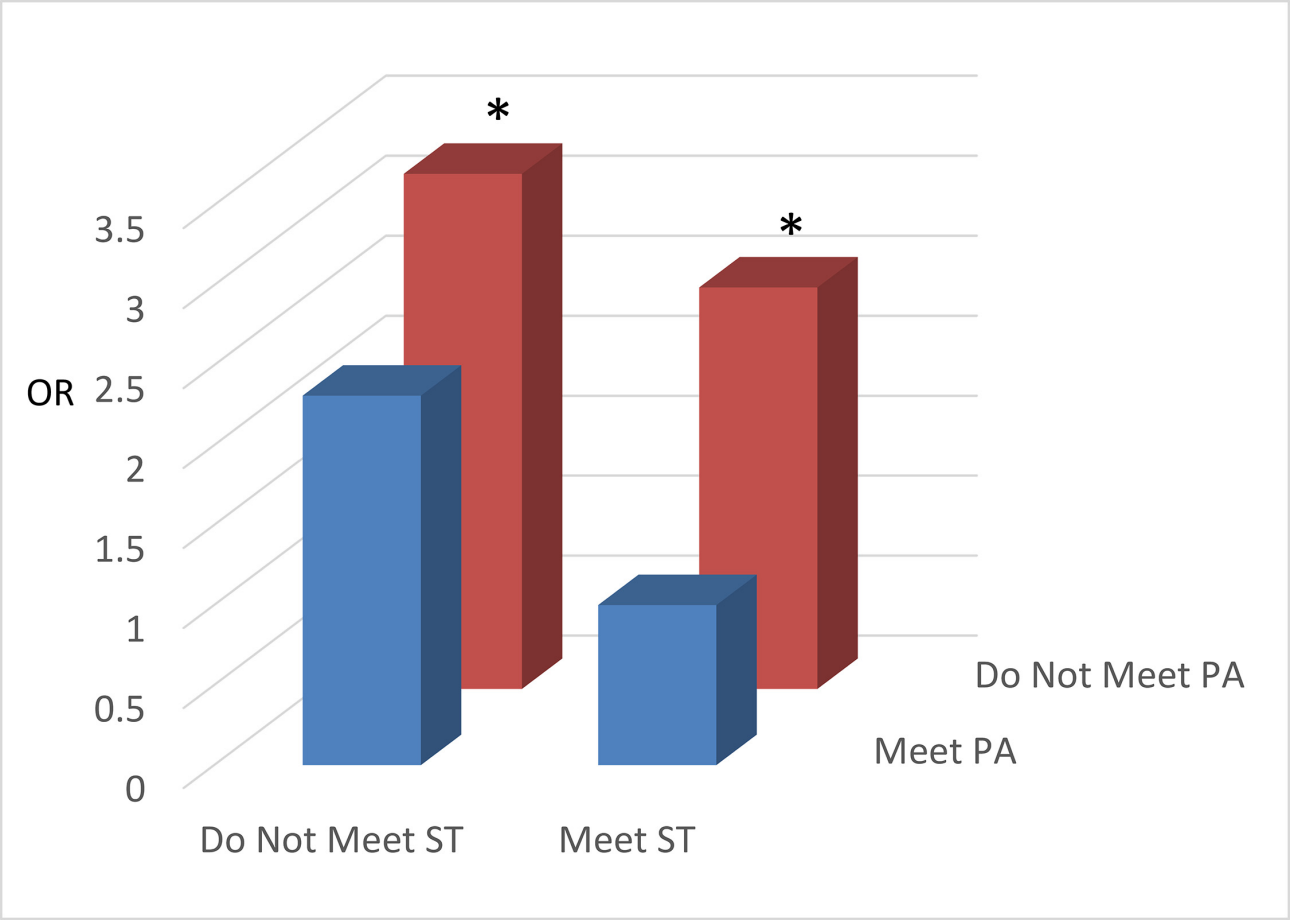
The joint associations of physical activity and screen time on cardiorespiratory fitness in adolescents. All the odds ratio were adjusted for age, gender, race/ethnicity, and socioeconomic status. * denotes significant association (P < 0.05) compared to the reference category (Meet PA/Meet ST). Abbreviations—OR: Odds Ratio; PA: Physical Activity; ST: Screen Time.

**Fig 3:**
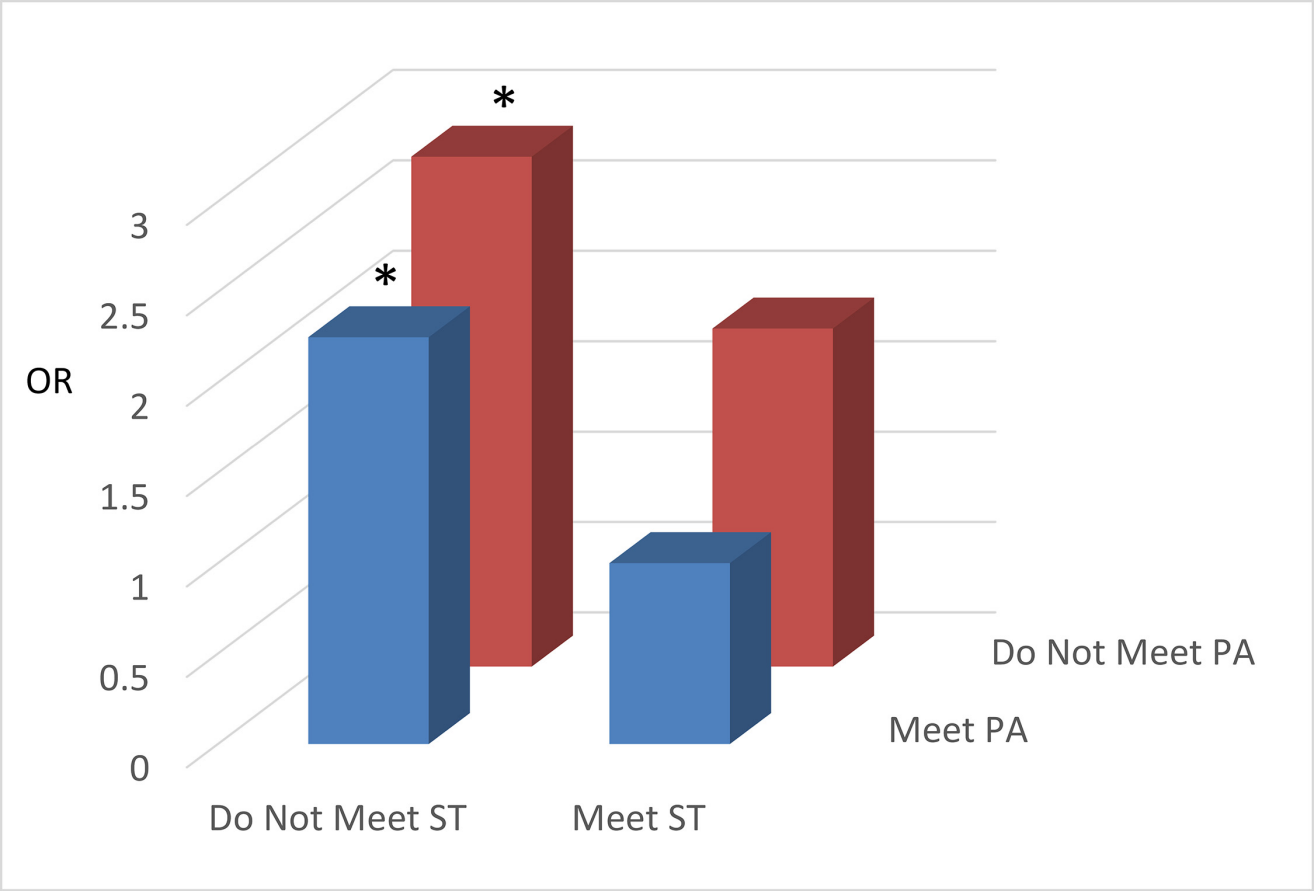
The joint associations of physical activity and screen time on overweight/obesity in adolescents. All the odds ratio were adjusted for age, gender, race/ethnicity, and socioeconomic status. * denotes significant association (P < 0.05) compared to the reference category (Meet PA/Meet ST). Abbreviations—OR: Odds Ratio; PA: Physical Activity; ST: Screen Time.

The results for the joint associations of meeting PA and ST guidelines with weight status and CRF are displayed in Fig 1 (children; only weight status is available) and Figs 2 and 3 (adolescents). Children not meeting either of the PA or ST guidelines had higher odds of being OW/OB than those in the reference group (meeting both guidelines). For adolescents, the odds of having low CRF was higher in the two ‘*Not Meeting PA Guideline*’ groups regardless of meeting ST guidelines (Fig 2). The odds of being OW/OB were considerably higher in the ‘not meeting ST guideline’ groups regardless of meeting PA guidelines compared with the referent group (Fig 3).

## Discussion

This study systematically evaluated the associations of PA and ST with weight status and CRF using nationally representative data in children and adolescents. A number of studies have explored these issues with both cross-sectional [29–32] and longitudinal designs [33] but this is the first study to report outcomes with this NNYFS sample. The results demonstrated that higher level of PA were associated with CRF, independent of ST in adolescents, while higher level of ST were associated with weight status, independent of meeting the PA guideline (or not) in children and adolescents.

The study helps to clarify factors influencing CRF in youth. We observed independent effects of PA on CRF in adolescents after controlling for ST, regardless of how PA was measured (e.g., daily METmin or numbers of days with at least 60 minutes). The results are consistent with a previous study [34] showing that ST did not affect CRF if a person was obtaining sufficient PA. Similarly, Denton et al. found that different intensities of PA, especially vigorous PA, were positively associated with CRF but not with sedentary time among youth (10–14 years old) using objectively measured PA and sedentary behavior as well as cycle ergometer maximal CRF test [20]. Moore et al. [21] found that both vigorous PA and sedentary time were independently associated with CRF, although sedentary time was on the borderline of being statistically significant (95% CI was 0 to 0.03). Our results build upon previous research indicating that there is limited evidence of adverse effects of excessive sedentary time or ST on CRF if youth are physically active. Our results differ from a few studies that reported significant associations of ST and/or sedentary behavior with CRF [22, 35]. The disparate findings may be due to differences in samples and the measures that were used. Therefore, the independent associations of ST and/or sedentary behavior with CRF needs to be further explored.

We found that both children and adolescents meeting the ST guideline had significantly better weight status regardless of meeting the PA guideline. Costigan et al. [5] reviewed 18 studies that examined the association of ST and weight/body composition from which only half (i.e., 9 studies) of the studies controlled for PA. Strong evidence (8 out of the 9 studies) suggested that screen-based sedentary behaviors were associated with weight status. Consensus has been achieved that screen-based sedentary behavior is more detrimental than other forms of sedentary behaviors such as playing board games, reading, or writing. This is likely due, in part, to two reasons; first, some screen-based sedentary behaviors (e.g. TV viewing) are often associated with poor diet habit, such as the increased consumption of unhealthy snacks, food, and high-energy drinks, [36, 37]; And second, ST may result in lower energy expenditure relative to other forms of sedentary behavior [38, 39]. This can also help to explain why the weaker or null independent association of overall sedentary behaviors and weight status were reported after controlling for PA [14, 33].

The joint influence of meeting PA and ST guidelines was supplemental to the independent analysis results. The findings were also consistent with previous studies in similar age groups despite the use of slightly different measures and methods across the studies. [14, 21, 40]. For example, Maher et al. [41] observed that meeting the ST guidelines is more important than meeting the 60 minutes PA guidelines in predicting overweight, especially among boys. In addition, Laurson et al. [40] reported that meeting the ST guideline was associated with being normal weight regardless of meeting the PA guidelines in girls. Although significantly higher OR of being overweight were observed among boys who did not meet both PA and ST guidelines, non-significant but higher OR were reported for meeting the ST guideline but not for meeting the PA guideline. The relatively small sample in that category could be the reason for non-significant findings. Moreover, our study indicated that meeting the PA guideline is more important to having healthy CRF levels opposed to meeting the ST guidelines. This could be explained by basic human physiology, where accumulating adequate amounts of PA, particularly MVPA, helps to maintain and/or improve CRF. Whereas simply reducing ST alone does not support or improve CRF.

Although the adoption of self-report measures in PA is more susceptible to measurement error compared to objective measures, the compliance to PA guidelines reported in the current study is reasonable and comparable. A total of 59.3% children and 27.5% adolescents indicated that they achieved the PA guidelines. Morrow et al. [42]reported a slightly higher compliance rate (~35%) achieving the PA guidelines in an adolescent sample and similar compliance (36%) was found in a smaller sample [43]. Considering the widespread use of the self-report measure in large-scale surveillance studies (e.g., NHANES, Youth Risk Behavior Surveillance System), it is important to provide the updated PA guideline compliance in both children and adolescent with a national representative sample to public health professionals and education practitioners. Not surprisingly, the compliance of meeting both PA and ST guidelines are low among children (i.e., 27.8%) and adolescents (i.e., 9.7%) in the current study. PA and ST may have different underlying mechanism toward weight status and CRF in youth. Public health agendas should target increasing PA and decreasing ST simultaneously, considering the low guideline compliance, particularly when it comes to meeting both guidelines concurrently.

Key strengths of the study include the nationally representative nature of the sample and the evaluation of both the independent and joint effects of PA and ST on weight status and CRF. However, the study is not without limitations. First, PA was measured by questionnaires, which is known to be more susceptible to measurement errors and reporting bias than objectively measured PA. Secondly, while several essential covariates were statistically controlled (e.g. age, gender, SES, and ethnicity), we were not able to control for some other factors (e.g. genetic variation, dietary habits, and/or parent influence) that could potentially confound the associations of PA and ST with weight status and CRF in youth. Lastly, the cross-sectional design prevents us from drawing causal inferences about youth behaviors and health-related fitness. Additional research is needed to examine the PA and ST associations with health outcomes in youth with objectively measured PA and ST. Information on implications of CRF in children aged 6–11 is also needed to determine the association of PA and ST on CRF in the younger age group.

## Conclusions

In summary, the study used the most recent national representative youth fitness and activity data to expand our understanding of relations between youth behaviors and health-related fitness. The results highlight the importance of being physically active (e.g, meeting the PA guideline) in order to promote healthy level of CRF. However, prolonged ST exerted a stronger influence on weight status among children and adolescents. These results reveal the complexities of evaluating associations between behaviors and health outcomes in youth. It is essential for public health initiatives to continue to promote the importance of meeting PA and ST guidelines in youth, ideally with an emphasis on meeting both guidelines concurrently.
